# Rare but relevant: a comprehensive narrative review of rare HIV-1 group M subtypes

**DOI:** 10.1128/mbio.01264-25

**Published:** 2025-08-21

**Authors:** Dana F. Indihar, Rachael Oke, Rondel Hemerding, De'Travean Williams, Rupert England, Laricca Y. London

**Affiliations:** 1 Department of Biological Sciences, Alabama A&M University, Huntsville, Alabama, USA; Albert Einstein College of Medicine, Bronx, New York, USA

**Keywords:** human immunodeficiency virus, rare subtypes, multidrug resistance, phylogenetic analysis, genetic recombination

## Abstract

Human immunodeficiency virus type 1 (HIV-1) group M comprises 10 genetically diverse subtypes, with subtypes C and B being the most prevalent globally. However, rare subtypes F, H, J, K, and L, though individually responsible for less than 1% of HIV-1 infections worldwide, play relevant roles in viral evolution, global persistence, recombination, and drug resistance. This review provides a comprehensive synthesis of the available literature on these rare subtypes for the first time, emphasizing their distribution, recombination patterns, transmission dynamics, and drug resistance-associated mutations. A key observation is that many of these rare subtypes are more frequently found in recombinant forms than in non-recombinant forms, which may expand their geographic distribution and sustain their epidemiological presence. We further highlight drug resistance-associated mutations in the protease and reverse transcriptase regions of the rare subtypes, which may affect treatment outcomes. Advances in molecular tools, such as next-generation sequencing and Bayesian phylogeographic analyses, have improved the identification of the rare subtypes in both recombinant and non-recombinant forms. However, there remains a significant data gap in the clinical impact of these subtypes as they continue to be undersampled and understudied. This review thus underscores the need for augmenting subtype-specific surveillance strategies, especially in regions where the rare subtypes predominantly circulate. Expanding research on these rare subtypes will be essential for understanding HIV-1 recombination and evolutionary dynamics, identifying trends in drug resistance, and developing more globally inclusive anti-viral strategies that better reflect the diversity of HIV-1.

## INTRODUCTION

Human immunodeficiency virus type 1 (HIV-1) is a zoonotic virus that originated from the Democratic Republic of Congo (DRC) around the 1920s (1–3). HIV-1 has four distinct lineages, each arising from a separate cross-species transmission event: the Major (M), New (N), Outlier (O), and P lineages. Group M originated from Kinshasa, the current capital of DRC, and is the lineage of most HIV-1 strains globally (1, 4). One or a few ancestral group M strains diverged from Kinshasa in the 1950s (1) into the 10 subtypes (and nine sub-subtypes) recognized today: A (A1–A4 and A6–A8), B, C, D, F (F1 and F2), G, H, J, K, and L (5, 6). Co-circulation of multiple subtypes within the same risk groups creates opportunities for co-infection, giving rise to circulating recombinant forms (CRFs). CRFs are mosaic viral strains comprised of genomic regions from two or more subtypes, identified in at least three epidemiologically unlinked individuals (7, 8). The non-recombinant or “pure” group M (sub-)subtypes were identified through sequencing data, either by sequencing the complete viral genome or individual genes (*env*, *gag*, and/or *pol* in particular), and then phylogenetically assessed for genetic relatedness (9). The classification of these distinct (sub-)subtypes required at least three “pure,” near-full-length viral genomic sequences that clustered equidistantly from other (sub-)subtypes, with a “star-like” (instead of “tree-like”) phylogeny (4). Subtypes E, I, and sub-subtype A5 have only been detected in recombinant viruses, precluding their classification as official group M (sub-)subtypes (4, 10, 11).

Subtype C is the most prevalent group M subtype, followed by subtypes B, A, G, and D (12, 13). In contrast, subtypes F, H, J, K, and L are the rarest, each responsible for approximately 1% or less of all HIV-1 infections globally (13). HIV-1 subtypes have been shown to differ in their response to antiretroviral therapy (ART), drug resistance, and pathogenesis (14). Most HIV-1 research has historically focused on subtypes B and C, but the rare subtypes contribute significantly to the persistence and genetic diversification of the virus worldwide. These rare subtypes remain epidemiologically and evolutionarily relevant due to their involvement in CRFs and the presence of ART resistance-associated mutations within their genomes. Despite their relevance, the scientific literature available on these rare subtypes is limited. Previous review articles written about HIV-1 subtypes primarily focus on the prevalence and distribution of common subtypes (e.g., subtypes A, B, and C), with only cursory mentions of the rare subtypes. Furthermore, these reviews often omit critical topics such as transmission dynamics, recombination patterns, and drug resistance profiles, which are essential for informing subtype-specific anti-viral strategies.

This review addresses an information gap in the HIV-1 field by offering the first comprehensive narrative overview of the available literature on these rare HIV-1 group M subtypes. It provides an updated overview of their classification and distribution, transmission dynamics, and recombination patterns and highlights their ART resistance-associated mutations that may affect therapeutic outcomes. These and other rare HIV-1 subtype characteristics are summarized in Table 1. By consolidating current knowledge on these underrepresented subtypes, this review emphasizes the ongoing contributions of rare subtypes to global HIV-1 diversity and ART resistance. This review also highlights the need for expanded research efforts focused on these rare subtypes, which is critical for anticipating future recombination trends, understanding HIV-1 evolution, and developing more targeted and efficient anti-viral strategies.

**TABLE 1:** Summary of rare HIV-1 subtype characteristics

Characteristics	Subtype F	Subtype H	Subtype J	Subtype K	Subtype L
Year first identified as potential subtype	1993	1994	1995	2000	2002
Year of subtype classification	1994	1994	1999	2000	2019
Sub-subtypes?	F1 and F2	No	No	No	No
Major countries of circulation today	F1: Angola, Romania, Italy, Spain, Brazil, Argentina, Belgium, DRC F2: Cameroon	DRC, Angola, Cameroon	DRC, Angola, Cameroon	DRC, Cameroon	DRC?
Known risk group association	F1: heterosexual, MSM, IDU, nosocomial, mother-to-child F2: potentially heterosexual	Heterosexual, mother to child	Heterosexual, mother to child	Unknown	Mother to child
Drug resistance mutations (RT region)	F1: M184V, T69D, L74I, Y188C, G190A, K103N/S F2: unknown	K65E, M184V, K103N/S	M184V, K103N/S	Unknown	Unknown
Drug resistance mutations (protease region)	F1: Q58E, L10V, M36I, M46I, I54V, V82A/I F2: M36I, M46I, L10V	M36I, K20R, V82A/I	L10V, M36I, M46I	Unknown	Unknown
CRFs?	Yes	Yes	Yes	Yes	Unknown
Example CRFs	CRF12_BF, CRF18_cpx variant	CRF27_cpx, A1H, CRF18_cpx, CRF04_cpx	CRF13_cpx, CRF06_cpx	CRF18_cpx, CRF06_cpx	Unknown
CRF drug resistance mutations	Multiple BF1 CRFs: G190A and K103N/S CRF18_cpx: I54V, M184V, G190A, K103N/S, V82A/I	K65E, M184V, K103N/S	CRF06_cpx: L10V, M36I, M184V, K103N/S	CRF18_cpx: I54V, M184V, G190A, K103N/S, V82A/I CRF06_cpx: L10V, M36I, M184V, K103N/S	Unknown

## ADVANCES IN RARE HIV-1 SUBTYPE DETECTION AND CLASSIFICATION

In the 1990s and early 2000s, when most of the rare subtypes were first discovered, subtype classification was determined by constructing phylogenetic trees using neighbor-joining or maximum-likelihood methods based on partial sequences from HIV-1 isolates, particularly from *env, gag,* and *pol* genes (4, 15–20). This approach was how subtypes F (15, 21), H (17, 22), J (18), and K (23) were originally identified. However, constructing phylogenetic trees from partial sequencing information was limited in its usefulness for distinguishing novel from recombinant subtype lineages. This reliance on partial sequencing data led to the erroneous classification of E and I as novel HIV-1 subtypes, which were later found to exist only in recombinant forms (4, 18). A minimum of three non-recombinant, near-full-length viral genomes is required for recognition as an independent group M subtype (4). In earlier eras, complete viral genomes of HIV-1 isolates were assembled by amplifying overlapping subgenomic fragments via PCR-based methods (23), but these methods could be error-prone and low throughput. Next-generation sequencing (NGS) transformed the detection of rare HIV-1 subtypes by enabling the high-throughput, full-genome sequencing of clinical viral isolates. The full-genome coverage facilitated by NGS enhances the detection of rare subtype sequences, recombination breakpoints, and subtype-specific drug resistance-associated mutations. For example, the classification of subtype L was only possible with metagenomic NGS that was combined with HIV-specific, target-enriched libraries to resolve contiguous genomic fragments and validate their distinctiveness from known group M subtypes (24). NGS remains critical for distinguishing recombinant and non-recombinant isolates and for detecting rare HIV-1 subtypes, particularly in the regions where they primarily circulate, such as Central and West Africa (12, 13, 25).

Advances in the fields of phylogenetics and phylogeography have also enhanced the detection and classification of rare subtypes. Tools such as SimPlot (26) use bootscanning and similarity plotting to identify recombination breakpoints in HIV-1 genomes. These tools, often in combination with reference sequence alignments and manual inspection of phylogenetic trees, improve detection of rare subtypes like K in both recombinant and non-recombinant forms (23). Bayesian Markov Chain Monte Carlo methods, as implemented in software such as Bayesian Evolutionary Analysis by Sampling Trees (BEAST), enable the temporal and spatial reconstruction of the evolutionary histories of HIV-1 lineages. Such methods have been applied to estimate the timing and geographic spread of rare HIV-1 subtypes. For example, BEAST was used to determine the timing and routes of introduction of subtype F from the DRC to Romania and South America (27), demonstrating how rare subtypes can be the source of major regional epidemics.

Bioinformatic subtyping tools have also been created to expedite subtype assignments. Among the most widely used subtyping tools are REGA, COntext-based Modeling for Expeditious Typing (COMET), Jumping Profile Hidden Markov Model (jpHMM), and Subtype Classification Using Evolutionary ALgorithms (SCUEAL). These tools compare user-provided query sequences to reference sequences, enabling the rapid and automated subtype classification and/or determination of recombination in HIV-1 genomic sequences. REGA assigns query sequences to subtypes or CRFs using phylogenetic tree-based classification (28). COMET is ideally suited for short genomic fragments and employs a probabilistic model (with hidden Markov chains) for rapid subtype or CRF assignment (29). The jpHMM tool aligns query sequences against subtype-specific profile HMMs to predict recombination breakpoints (30). SCUEAL integrates a maximum-likelihood multi-model inference approach with a reference sequence library for subtype assignment and inference of recombination events (31). Collectively, these tools have enabled the robust and rapid analysis of HIV-1 genomic sequences, becoming indispensable for viral epidemiological and evolutionary research.

In summary, the detection and classification of rare HIV-1 subtypes have advanced significantly through the integration of NGS and bioinformatic tools to sample and analyze HIV-1 genomes. These advances improve our understanding of the phylogenetic relationships, recombination patterns, and evolutionary dynamics of rare HIV-1 subtypes.

## SUBTYPE F

**Fig 1:**
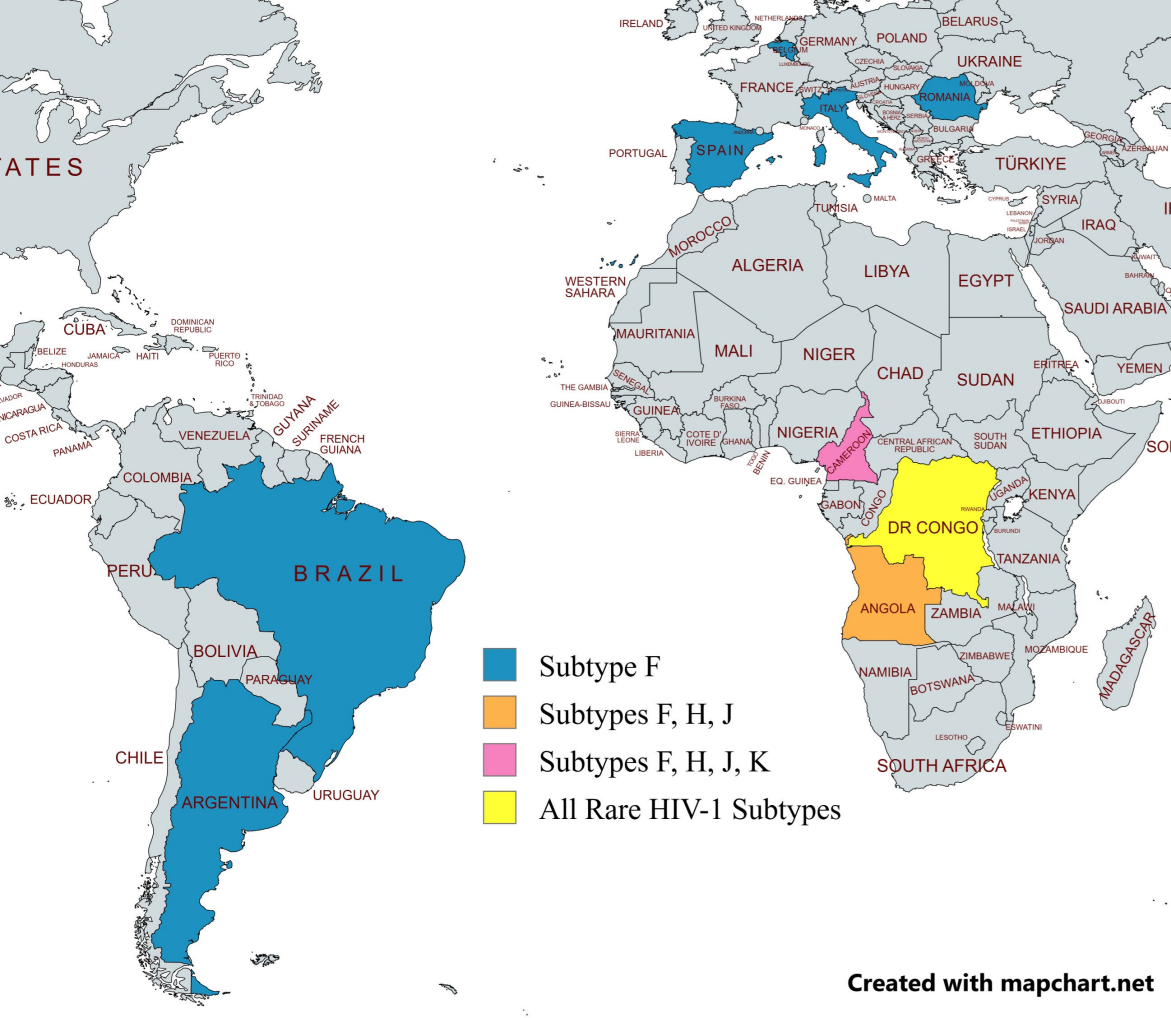
Major countries where the rare HIV-1 subtypes circulate today. This figure is a map depicting the main countries where the majority of non-recombinant, rare HIV-1 subtype strains circulate. The countries where only subtype F circulates are shown in blue (Brazil, Argentina, Spain, Belgium, Italy, and Romania). Angola, where subtypes F, H, and J primarily circulate, is depicted in orange. Cameroon, where all subtypes excluding subtype L circulate, is shown in pink. The DRC, where all the rare subtypes can be found, is highlighted in yellow. This figure was created using mapchart.net.

Subtype F is responsible for less than 1% of all HIV-1 infections globally (13) but remains a prominent subtype in some countries, including Romania (nearly 70% of HIV-1 cases [32]), Angola (8%–23% of infections [33]), and Brazil (10%–20% of cases [33]). The major countries where subtype F (and the other rare HIV-1 subtypes) primarily circulates today are shown in Fig. 1. It was first recognized as a potential subtype in 1993, following the phylogenetic analysis of 70 *gag* genes from an international pool of HIV-1 isolates (15, 21). A separate analysis comparing the *env* sequences from nine Romanian HIV-1 isolates to those of African and Brazilian isolates further confirmed the existence of this subtype in 1994 (16). In 1999, phylogenetic analysis of *env* and *gag* sequences from 11 Central African HIV-1 isolates (using the Kimura two-parameter method and unrooted neighbor-joining trees) suggested that subtype F comprised three sub-subtypes: F1, F2, and F3 (20). However, additional analysis of the near-full-length genomes of six of those isolates in 2000 led to the reclassification of F3 as a distinct subtype, subtype K (described in section Subtype K). The F1 and F2 isolates were shown to be non-recombinant viruses, and the genetic distances between them were low (23). This implied that the sub-subtypes shared a common ancestor and had diverged from each other recently (23, 34). Although it is not known when F1 and F2 evolved into separate sub-subtypes, an analysis of mixed-effects molecular clock models used to estimate divergence times suggested that subtype F may have emerged from Central and West Africa between the 1940s and 1960s (34).

F1 is more prevalent than F2 globally, both in recombinant and non-recombinant forms, and presently circulates in Africa, South America, and Europe (35). According to phylogeographical analyses, which were conducted using BEAST v.1.7.4 software and the Bayesian Markov Chain Monte Carlo approach, F1 spread from the DRC to Angola in the 1950s and to South America between the 1950s and 1970s, from Angola to Romania in the 1960s, and then from South America and Romania to Italy in the 1970s (27). F1 continued to spread throughout Africa, Europe, and South America in the 1980s and 1990s, likely due to international migration and travel (33, 35). F1 may have expanded from Angola due to heterosexual transmission during the independence war and subsequent civil war in 1975, which triggered emigration from Angola and the arrival of foreign troops from multiple nations (33, 35), including Romania, potentially introducing F1 to Romania (33). This idea is supported by the close phylogenetic relationship between Angolan and Romanian F1 strains (36). In Romania, F1 was initially spread among adults via heterosexual transmission, but by 1989, a pediatric epidemic emerged through nosocomial routes, including from unscreened blood transfusions and reused needles and syringes (32, 33, 37). In the 1980s and 1990s, Romanian strains contributed to the spread of F1 to Western Europe, particularly Italy, where phylogenetic analyses have traced Romanian and Italian F1 sequences to a common origin (27). The F1 epidemic in Brazil may have arisen from a single introduction event around 1970 from an African-origin strain. Travel and migration from Brazil to Europe in the 1990s and 2000s, especially to Spain and the UK, likely contributed to the expansion of F1 in Europe during this period (27, 35). Although heterosexual transmission accounts for approximately 70% of F1 infections (27), F1 has been documented in a large variety of transmission networks, including men who have sex with men (MSM) (27, 32, 33, 38–43), injection drug use (IDU) (32, 33, 44, 45), mother to child (46, 47), and nosocomial pediatric infections (32, 33, 37). Circulation of F1 within MSM networks may constitute a distinct HIV-1 epidemic relative to other risk groups (40) and could contribute to the perpetuation of HIV-1 in European and South American countries, including Spain (38, 41, 43), Italy (27), Argentina (40), Belgium (39, 42), and Brazil (33). Over time, F1 has diverged into two major strains: F1.I (found predominantly in Brazil) and F1.II (found mostly in Europe and Africa) (35). This bifurcation may be a result of genetic drift acting on founder strains that emerged through geographic isolation and were then shaped by selective pressures and local transmission dynamics.

F2 appears to be primarily restricted to Central Africa (48), with most full-length non-recombinant isolates and F2-containing CRFs originating from Cameroon (5). Other F2 genomic sequences have been identified in Belgium (42), Japan (49), and the UK (50), but it is unclear whether those sequences originated from recombinant or “pure” F2 isolates. It is not known why F2 remains largely confined to Central Africa while F1 has expanded globally. There is no evidence in the current literature to suggest that F2 harbors mutations that reduce its transmissibility. Moreover, no studies have directly characterized the transmission dynamics of F2. However, its restriction to Central Africa suggests that it is primarily transmitted through heterosexual contact, as that is the predominant mode of HIV-1 transmission in that region (51, 52). Notably, heterosexual contact has been shown to be less efficient at transmitting HIV-1 (per exposure) compared to other routes such as receptive anal intercourse or injection drug use (53–55). This may be a contributing factor to the limited geographic spread of F2, especially when compared to F1. The broader transmission versatility of F1 compared to F2 may thus explicate their differing global distributions.

Most subtype F CRFs are recombinants of sub-subtype F1 and subtype B (5), which suggests a high frequency of dual infections in populations where both B and F1 circulate (56). These CRFs are found primarily in Brazil (45, 56–67), Argentina (68–73), and Spain (66, 72, 74–78). Shortly after F1 was introduced to South America, it recombined with subtype B to form a CRF12_BF-like variant, which gave rise to additional BF CRFs with similar recombination breakpoints and contributed to the evolution and spread of F1 in the region (79). Notably, virologic response rates to ART have been reported to be significantly lower in individuals infected with subtype F compared to those infected with subtype B (38), though the reasons for this are unclear. Both non-recombinant and recombinant forms of subtype F harbor mutations associated with reduced ART efficacy, particularly within their protease and reverse transcriptase (RT) gene regions. Protease inhibitor (PI)-associated mutations were found in subtype F strains from Guinea (80), Romania (81–83), Spain (84), Central Africa, and France (85). These include Q58E (80), L10V (84, 85), M36I (81, 84, 85), M46I (82, 85), I54V (82, 83), and V82A/I (82, 83). M36I, M46I, and L10V may be the most widespread, as they have been found in both F1 and F2 sequences from Central Africa (85) and in F1 strains from Spain (M36I [84] and L10V [84]) and Romania (M36I [81] and M46I [82]). RT mutations associated with resistance to nucleoside reverse transcriptase inhibitors (NRTIs) (M184V [47, 82–84], T69D [82], and L74I [82]) and non-nucleoside reverse transcriptase inhibitors (NNRTIs) (Y188C [82], G190A [47], and K103N/S [47, 64]) were observed in subtype F strains from Romania (82, 83), Brazil (47, 64), and Spain (84). The M184V mutation was identified in F1 strains from all three countries (47, 82–84), along with the G190A and K103N/S mutations in multiple BF1 CRFs (47, 64). Interestingly, the South African CRF18_cpx variant (a complex recombinant of subtypes F, A1, G, H, K, and some undefined subtypes) also carries V82A/I, I54V, M184V, G190A, and K103N/S mutations (14), indicating a resistance profile with similarities to subtype F. The presence of ART resistance mutations in both recombinant and non-recombinant subtype F strains suggests that they may be reservoirs for ART-resistant HIV-1 variants, highlighting the continued need to monitor subtype-specific resistance patterns for optimizing anti-HIV treatment strategies.

## SUBTYPE H

Subtype H accounts for less than 1% of HIV-1 infections globally (13) and exhibits the slowest evolutionary rate of all group M subtypes, as estimated by mixed-effects molecular clock models (34). It was initially proposed as a distinct subtype in 1994 following its recognition in viral isolates from Kenya (22) and Central Africa (17, 86, 87). The *env* regions of these isolates clustered separately and equidistantly from the *env* regions of other group M subtypes (17, 22). Ancestral lineages of subtype H may have been seeded in Matadi from Kinshasa between 1950 and 1960, and due to the poor connection of Matadi to other African cities, the transmission of subtype H was restricted to the DRC and Northern Angola (88). Some of the earliest isolated cases of subtype H reported outside of Africa were in Belgium (89) and London (90); however, the infected patients in both cases had previously lived in the DRC (89, 90). The global proportion of subtype H infections has significantly decreased over the past decade (13), but it continues to primarily circulate in Central, Southern, and Western Africa today (12, 13, 25).

No studies to date have explicitly investigated the transmission dynamics or risk groups associated with subtype H. Nevertheless, subtype H has been documented in cases of both mother-to-child (22, 91, 92) and heterosexual (90) transmission. It is unclear whether the pathogenesis of subtype H differs between these two risk groups. Subtype H is also the recombination parent of at least 10 CRFs archived in the Los Alamos National Laboratory (LANL) HIV Sequence Database, recombining most frequently with subtype A (5). These CRFs have been found globally, including in the DRC (e.g., CRF27_cpx [recombinant of H, A, E, G, and unclassified segments]) (93, 94), Cameroon (e.g., A1H) (95, 96), Cuba (e.g., CRF18_cpx) (97), and Greece (e.g., CRF04_cpx [recombinant of H, A1, G, and unclassified segments]) (98, 99). Despite its rarity, the widespread geographic distribution of subtype H-containing CRFs suggests that recombination is critical for subtype H circulation outside of Africa, and these CRFs may play a crucial role in shaping the global HIV-1 recombination landscape.

Both recombinant and non-recombinant subtype H isolates have been identified with mutations associated with ART resistance. The M36I mutation is the most frequently observed PI-associated mutation for subtype H and has been reported in isolates from Cuba (100), Cameroon (101), and within an international panel of subtype H isolates (102). The K20R (101) and V82A/I (102) PI-associated mutations have also been found in this subtype. NRTI-associated mutations have been observed in both non-recombinant and recombinant subtype H strains, including K65E (103) and M184V (104), respectively. Both forms of subtype H have also been shown to harbor the K103N/S mutation, which is associated with high-level resistance to NNRTIs (90, 104). Interestingly, multiple studies have demonstrated that subtype H exhibits hypersensitivity to NNRTIs (105–107), suggesting that NNRTI-based regimens may be effective against this subtype when resistance mutations (such as K103N/S) are not present. These findings further underscore the importance of considering HIV-1 subtype when devising ART treatment strategies.

## SUBTYPE J

Subtype J accounts for approximately 0.1% of all HIV-1 infections globally (13, 108). As estimated by mixed-effects molecular clock models, subtype J evolves at the second lowest rate of all group M subtypes (34). It was first recognized as a potential new subtype in 1995 following the phylogenetic analysis of representative sequences from subtypes A–H and HIV-1 *env* and *gag* sequence fragments isolated from blood samples collected in Sweden. The samples came from two unrelated HIV-infected immigrant patients from the DRC (18). In 1999, fresh blood samples obtained from the same patients were used to clone and sequence the first near-complete proviral genomes of two primary subtype J isolates. The isolates were non-recombinant and clustered both independently and equidistantly from reference subtype A–H sequences, supporting the classification of those isolates as part of a distinct subtype (19). In 2005, genomic analysis of subtype J fragments within CRF13_cpx (recombinant of J, A1, G, CRF01_AE, and unclassified segments) isolates revealed low branching indices and similar divergence patterns to those observed between established sub-subtypes such as F1 and F2, suggesting that subtype J may comprise distinct lineages warranting its reclassification into sub-subtypes J1 and J2 (109). However, subtype J has yet to be subdivided, likely due to the limited number of full-length non-recombinant genomes and insufficient intra-subtype branching required to meet the established sub-subtype classification criteria (4).

Similar to subtype H, subtype J may have been transported from Kinshasa to Matadi between 1950 and 1960, and the seclusion of Matadi may have limited early subtype J transmission to individuals in the DRC and Angola (88). This idea is supported by recombinant and non-recombinant subtype J isolates obtained from Angolan patients in 1993, suggesting that Angola was an early origin of the subtype (110). Today, non-recombinant subtype J remains geographically restricted to West and Central Africa (12, 13), with most isolates coming from the DRC (18, 111), Angola (88), and Cameroon (112). Due to its rarity, there is no evidence in the current literature identifying a specific population or risk group (e.g., MSM or IDU) disproportionately affected by subtype J, nor have there been reports of distinct virological characteristics or clinical outcomes associated with this subtype (relative to other group M subtypes). Although the transmission modes for subtype J are not well-characterized, its transmission through both heterosexual contact (18, 113, 114) and mother to child (111) has been documented. The low transmission efficiency and high recombination potential of subtype J have been hypothesized as reasons for its limited epidemic success globally in non-recombinant forms (19, 110).

Although the global prevalence and geographic range of subtype J are limited, recombination likely contributes to the expansion of this subtype outside of Africa. CRFs containing subtype J as a recombination parent circulate more widely than “pure” subtype J strains. While most subtype J-containing CRFs have been isolated from Cameroon (5), CRFs such as CRF06_cpx (a recombinant of subtypes J, A, G, and K) have been found in China (115), the UK (116), and Australia (117). A unique H/J recombinant has also been reported in Canada (118). Nonetheless, subtype J appears less frequently as a parental lineage in CRFs than more common subtypes such as A or C. Both CRF06_cpx and non-recombinant subtype J isolates have been shown to harbor similar drug resistance-associated mutations, including PI-associated mutations (L10V and M36I [85, 101, 119]), the NRTI-associated mutation M184V (120, 121), and the NNRTI-associated mutation K103N/S mutation (121, 122). The M46I (120) PI-associated mutation has also been identified in non-recombinant subtype J isolates. However, the impact of these mutations on ART resistance among individuals infected with “pure” subtype J remains poorly understood. This scarcity of epidemiological data (in addition to the limited availability of full-length subtype J sequences) highlights the need for increased genomic surveillance in undersampled regions like Central and Western Africa (123), where rare subtypes like J may be circulating undetected.

## SUBTYPE K

Subtype K was originally classified as sub-subtype F3 (20) until it was reclassified as a distinct subtype in 2000 (23). The first two strains used to identify this subtype, 97ZR-EQTB11 and 96CM-MP535, were isolated from the DRC and Cameroon, respectively, in the late 1990s (20, 23). Near-complete genomes of the two strains were sequenced by amplifying overlapping gene regions with PCR, followed by Sanger sequencing and assembly into consensus sequences for analysis. Subsequent comparative analyses using phylogenetic tree construction, diversity plotting, and bootscanning were conducted, and the two genomes were shown to cluster separately and equidistantly to all other group M subtypes (23). VI354, a strain isolated from an AIDS patient in Gabon in 1989 (124), clustered with 97ZR-EQTB11 and 96CM-MP535 (23), fulfilling the criteria required for those strains to be classified as a new subtype (4, 23).

“Pure” subtype K strains are extremely uncommon. They appear to be restricted to Central Africa, but the last one documented in the LANL HIV Sequence Database (5) was isolated in 1997 (6). This rarity suggests that subtype K may not be as transmissible as other group M subtypes (125) and raises questions about whether “pure” subtype K still circulates. Most genetic sequences identified as subtype K today exist within recombinant forms (126). Subtype K is a recombination parent for at least 15 CRFs in the LANL HIV Sequence Database (5), and these CRFs are pervasive globally. One example, CRF18_cpx, has been detected in South Africa (14), Cuba (97), and the UK (116). Another example, CRF06_cpx, was shown to circulate in several West African countries (Senegal, Mali, Nigeria, etc.) (127) where it still circulates today (128) and recently in the UK (116), Cyprus (129), and China (115). Notably, subtype K recombines most frequently with subtype A, a trend observed in multiple CRFs (126), including CRF18_cpx (14) and CRF06_cpx (127). Recombination thus appears to be the primary driver of subtype K’s continuation, suggesting that recombination, rather than simple lineage persistence, may be the reason for the “survival” of this subtype.

Independent subtype K infections are rare, and there is little evidence of non-recombinant subtype K infections in the literature after 2000 (23). Thus, there is limited information regarding transmission patterns or risk groups linked with this subtype. However, CRFs with subtype K as a recombination parent have been isolated primarily from heterosexual individuals (14, 97, 115, 127–129) and MSM (97, 116, 128, 129). These CRFs, particularly CRF18_cpx (described in section Subtype F), have been shown to contain drug resistance-associated mutations and to be multidrug-resistant in isolated cases (14, 85, 130–132). The presence of subtype K in recombinant strains with ART resistance-associated mutations may thus contribute to treatment failure, although its specific effect on resistance mechanisms remains unclear. Efforts to elucidate the role of subtype K in ART resistance are further complicated by the availability of complete subtype K genomes. The most complete publicly available genomes, those of 97ZR-EQTB11 and 96CM-MP535, are both missing their long terminal repeat (LTR) extremities (23), and no other subtype K LTR sequences have been archived in the LANL HIV Sequence Database (5). This limits investigations into subtype K biology and its potential role in ART failure, particularly given that genetic variants in the LTR extremities of HIV-1 isolates have been shown to affect ART resistance both *in vitro* (133) and *in vivo* (134). Whether such variants exist in the subtype K LTRs remains unknown. Even in the absence of additional “pure” subtype K genomes, its presence in multidrug-resistant recombinant strains underscores the importance of characterizing its full genomic architecture (including the LTR regions) to understand its potential contributions to HIV-1 evolution and ART resistance globally.

## SUBTYPE L

The earliest known strain of what would later be classified as subtype L (83CD003) was first isolated in 1983 from an AIDS patient in Kinshasa, DRC (135, 136). A second strain (90CD121E12) was isolated in 1990 from a 12-month-old male in Kimpese, DRC (137, 138). The third and final known strain (CG-0018a-01) was isolated from Kananga, DRC, in 2001 (24, 25). In 2001, the evolutionary relationships of 83CD003 to over 200 group M HIV-1 isolates from the DRC were assessed by estimating pairwise nucleotide distances using the F84 model and the neighbor-joining method to construct an unrooted phylogenetic tree. The analysis revealed that 83CD003 did not cluster with any known subtype or unclassified isolates from the DRC, suggesting its viral lineage was either rare or extinct in the region (136). A 2002 phylogenetic analysis of 83CD003 and 90CD121E12 using the F84 model and bootstrap analysis showed that the two strains shared 95% genetic identity and formed a distinct cluster equidistant from subtypes A through K. However, the cluster was unable to be classified as a subtype until at least one additional isolate from the same lineage was found (138). CG-0018a-01 was isolated in 2001, but it was not recognized as a potential member of that cluster until 2017 (24, 25). RT-PCR followed by Sanger sequencing was conducted on the *env* immunodominant region (IDR) of HIV-1 samples collected from the DRC between 2001 and 2003. Only 1 out of the 172 *env* IDR sequences analyzed (CG-0018a-01) was identified as subtype L. The CG-0018a-01 *env* IDR was found to cluster basal with those of 90CD121E12 and 83CD003. However, it was impossible to determine if the CG-0018a-01 *env* IDR sequence originated from a “pure” subtype L isolate or from a CRF (25). After a complete genome for CG-0018a-01 was later generated by combining metagenomic NGS and HIV-specific target-enriched libraries, the CG-0018a-01 isolate was confirmed as a member of the same cluster as 90CD121E12 and 83CD003. The three strains formed a distinct evolutionary lineage separate from the other group M subtypes, and their divergence was not due to recombination. Thus, subtype L was able to be confirmed as the newest HIV-1 group M subtype in 2019 (24).

All three documented cases of subtype L originated from the DRC, with no confirmed reports of the subtype outside of that region (24, 25, 135–138). To date, confirmed partial or complete subtype L sequences have been restricted to the DRC, and no additional sequences have been archived in the LANL HIV Sequence Database (5) since the early 2000s, which raises questions about the subtype’s current circulation and existence. The absence of detected subtype L strains outside the DRC does not necessarily indicate that the subtype is restricted to that region, as it may be present in other areas with limited sequencing capacity. The small number of confirmed cases confined to the DRC thus suggests either limitations in surveillance efforts for detecting subtype L or that the subtype has remained geographically restricted due to epidemiological bottlenecks or low viral transmission fitness (24, 136, 138). Interestingly, there are no known CRFs that contain subtype L as a recombination parent. This may indicate that subtype L is either transmitted within isolated networks and/or that it circulates at a low prevalence, both of which would limit opportunities for recombination with other HIV-1 subtypes.

Due to the rarity of subtype L, there are insufficient data to determine its transmission patterns and risk groups. The 90CD121E12 strain was transmitted from mother to child (137, 138), but the transmission routes for 83CD003 and CG-0018a-01 are not known. The uncommonness of subtype L also causes it to be omitted from studies that compare clinical outcomes across different group M HIV-1 subtypes, which means that the pathogenesis of subtype L in comparison to other subtypes is poorly understood. Furthermore, there have been no reports of distinct clinical manifestations or ART resistance associated with this subtype. Its implications for treatment efficacy and vaccine development thus remain unknown. Since the last complete subtype L genome was isolated in 2001 (24, 25), its persistence in either “pure” or recombinant forms in the DRC or other regions is undetermined. This underscores a need for public health strategies that include genomic surveillance tools capable of detecting rare subtypes (such as NGS) to monitor the future spread of emergent or undersampled HIV strains. In the event of future subtype L detections, comprehensive documentation of its transmission circumstances, clinical presentation, and response to ART will be critical so that its impact on public health, especially in comparison to other subtypes, can be assessed.

## CONCLUSION

While the HIV-1 research landscape and ART treatment strategies primarily center on the globally dominant subtypes B and C, this review illustrates why the rare group M subtypes remain relevant. Their unique geographic distributions, transmission histories, and recombination potential contribute to our understanding of HIV-1’s complex evolution. Although subtypes F, H, J, K, and L each account for <1% of global infections (13), their presence in CRFs suggests that recombination sustains their epidemiological relevance worldwide. These rare subtypes (both in recombinant and non-recombinant forms) often contain mutations associated with ART resistance, including the K103N/S (14, 47, 64, 121, 122) and M184V (14, 47, 82–84, 104, 120, 121) mutations, which have been identified across multiple rare subtypes. The presence of these mutations raises concerns about the effectiveness and suitability of existing ART regimens in regions where these rare subtypes circulate, particularly Central and West Africa. ART regimens optimized for the treatment of subtypes B and C may demonstrate reduced efficacy against the rare subtypes, emphasizing the importance of developing region- and subtype-specific anti-viral strategies.

Although many of these rare subtypes are geographically restricted to regions with limited genomic surveillance infrastructure, their continued circulation highlights the importance of expanding surveillance and research efforts in the regions where they persist. These subtypes are consistently underrepresented in global data sets, obscuring their potential true epidemiological burden. Their pathogenesis, transmission dynamics, and ART resistance profiles are poorly defined, which may contribute to the underestimation of their role in drug resistance and recombination-driven diversity. Given that HIV-1 subtype affects ART treatment response, drug resistance, and pathogenesis (14), expanding research on these rare subtypes is essential. Advancing such research efforts may prove challenging in countries with limited infrastructure, financing, and access to the advanced genomic technologies necessary for sequencing and accurate subtype and CRF classification. Nevertheless, advances in phylogenetics, NGS, and subtype classification tools make it feasible to uncover new insights into these understudied subtypes. Leveraging these technologies will improve our understanding of HIV-1 evolution, enhance our ability to predict emerging resistance patterns, and inform the development of more broadly applicable therapeutic and preventive strategies that better reflect the global diversity of HIV-1.
